# Spatial Organization of Morpho‐Electric Subtypes of Pyramidal Neuron in the Subiculum

**DOI:** 10.1002/hipo.70081

**Published:** 2026-02-16

**Authors:** Alix Guinet, Joachim Behr, Imre Vida, Sabine Grosser

**Affiliations:** ^1^ Institute for Integrative Neuroanatomy Charité—Universitätsmedizin Berlin Berlin Germany; ^2^ Institute for Biology Humboldt ‐ Universität zu Berlin Berlin Germany; ^3^ Department of Psychiatry, Psychotherapy and Psychosomatic Medicine Brandenburg Medical School Neuruppin Germany; ^4^ Department of Psychiatry and Psychotherapy Charité—Universitätsmedizin Berlin Berlin Germany; ^5^ Faculty of Health Science Brandenburg, Joint Faculty of the University of Potsdam Brandenburg University of Technology Cottbus‐Senftenberg and Brandenburg Medical School Theodor Fontane Potsdam Germany

**Keywords:** discharge, hippocampus, intrinsic physiology, morphology, PCA, pyramidal neurons, subiculum

## Abstract

The subiculum is a main output structure of the hippocampus, transmitting information from the CA1 to the entorhinal cortex in a spatially structured manner. Prior studies revealed a high electrophysiological and molecular heterogeneity of subicular pyramidal neurons (PYNs) with evidence for further spatial subdivisions of the subiculum. In this study we focused on the cellular organization of the proximal to mid‐distal part of the subiculum, designated as subregion 2 (Sub2) and performed a comprehensive electrophysiological and morphological characterization of PYNs by whole‐cell patch‐clamp recordings combined with intracellular labeling in acute rat hippocampal brain slices. Principal component analysis based on discharge pattern‐related parameters and subsequent unsupervised, hierarchical clustering classified the PYNs into three subtypes: regular firing (RF), weak‐bursting (WB), and strong‐bursting (SB) neurons. Electrophysiological analysis revealed further differences between RF neurons and the two subtypes of bursting neurons in their active and passive properties. The three subtypes also showed differences in their morphometric features, including the apical and basal dendritic spread and branching pattern. Additionally, we identified a divergent morphological subset among RF neurons, bearing two apical dendrites. Mapping the three PYN subtypes onto the subiculum revealed specific spatial distributions along the superficial‐deep and proximo‐distal axes. Thus, this work maps the heterogeneity of subicular PYN onto differentially distributed subclasses in the Sub2 region with distinct physiological and morphological features. These findings together with prior observations of divergent anatomical projections from subicular subregions are pivotal for the understanding of how subicular morpho‐electric neuron types relate to each other and contribute to processing and distribution of information to cortical regions from this hippocampal output region.

## 1 Introduction

The subiculum serves as a major processing station between the hippocampus proper and the entorhinal cortex (EC). However, it also interacts with other cortical subregions with diverse functions. As an interface between CA1 and the EC, it modulates the transfer of information from and to the hippocampus. Inputs to the subiculum show a high level of spatial organization. Distal CA1 PYNs, adjacent to the subiculum, target the proximal subiculum, whereas proximal CA1 neurons project to the distal subiculum (Amaral et al. 1991), with information also being projected back from the subiculum to CA1 (Sun et al. 2014). The subicular output to the EC, as well as the reciprocal connections from the EC to the subiculum, are also spatially organized with the proximal subiculum being preferentially connected to the lateral EC and the distal subiculum to the medial EC (Naber et al. 2000; Witter 2006). Finally, subicular outputs to other cortical regions also exhibit a spatial organization, with the proximal subiculum projecting to the perirhinal, the prelimbic, and the infralimbic cortices, the nucleus accumbens, and the amygdala (Ding 2013; Witter 2006), versus the distal subiculum projecting to the retrosplenial cortex, the postrhinal cortex, and the presubiculum (Witter 2006). In contrast to CA1, subicular PYNs mostly target only one brain region in rats (Naber and Witter 1998). In addition to the information flow from and to various cortical areas, the subiculum displays a high level of intrinsic connectivity, as excitatory synapses and population activity could be shown in ex vivo subicular slices (Böhm et al. 2018).

To facilitate the integration and processing of this vast number of inputs and outputs from and to different cortical and subcortical areas, the subiculum requires a flexible intrinsic network of neurons exhibiting distinct functional and cellular properties. In fact, subicular PYNs have been classified into two subpopulations in terms of their discharge pattern. Regular firing (RF) neurons respond with trains of single action potentials (APs), whereas burst firing (BF) neurons fire two or more APs riding on a depolarizing envelope (burst) at the onset of a depolarizing current pulse (Stewart 1997). This divergence in firing pattern is complemented by reports of further differences in electrophysiological and morphological properties (Behr et al. 1996; Graves et al. 2012; Kim and Spruston 2012; Staff et al. 2000; Taube 1993), suggesting that subicular PYNs may comprise morpho‐electric subtypes, but findings have been contradictory. Moreover, the proportion of RF to BF neurons has been found to change along the proximo‐distal axis of the subiculum (Cembrowski, Phillips, et al. 2018; Cembrowski, Wang, et al. 2018; Jarsky et al. 2008; Kim and Spruston 2012; Menendez de la Prida 2003), but PYN heterogeneity has not been studied over the full extent of subiculum 2.

In addition, recent studies revealed anatomical subdivisions based on the presence of specific neuronal markers such as fibronectin (FN1) and calbindin (CB) as well as cytoarchitectonics (Fujise et al. 1995; Ishihara and Fukuda 2016; Ishihara et al. 2020). Ishihara and Fukuda identified two subregions within the subiculum: subiculum 1 (Sub1) and subiculum 2 (Sub2). Sub2 is situated proximally to CA1, extending into the mid‐distal part, and displays a laminar structure of pyramidal neurons exhibiting larger somata. In contrast, the distal Sub1 is a core‐like structure of densely packed PYNs with smaller cell bodies (Ishihara et al. 2020). However, how electrophysiological and morphological properties of subicular PYNs can be mapped onto the subiculum and its subregions is not fully understood.

Here we focus on Sub2, which has a laminar organization and investigate neuronal heterogeneity and its distribution along the proximo‐distal and superficial‐deep axis in this area.

## 2 Materials and Methods

All experiments were conducted in agreement with the European Council Directive 86/609/EEC, the German Animal Welfare Act and guidelines from local authorities (Berlin, T‐CH 0020/40). The dataset analyzed here included 26 neurons from a previously published study (Guinet et al. 2024), in which we focused on inhibitory synaptic transmission and plasticity. Of these neurons, additional physiological parameters were obtained, and a more detailed morphological characterization was performed. An additional 122 neurons were recorded and analyzed for the current study.

### 2.1 Preparation of Acute Brain Slices

Acute horizontal hippocampal brain slices were obtained from juvenile (P20–30), male and female VGAT‐Venus Wistar rats (Uematsu et al. 2008). Although not fully mature, juvenile brain tissue provides optimal viability and accessibility for stable, reproducible electrophysiological measurements, which makes this developmental stage optimal for whole‐cell patch‐clamp recordings. After deep isoflurane anesthesia (3%), animals were decapitated, and brains were quickly removed. Brains were transferred to semi‐frozen, carbogenated, sucrose‐based artificial cerebrospinal fluid (sACSF) containing (in mM): 87 NaCl, 2.5 KCl, 25 NaHCO_3_, 1.25 NaH_2_PO_4_, 25 Glucose, 75 Sucrose, 1 Na_2_‐Pyruvate, 1 Na_2_‐Ascorbate, 7 MgCl_2_, 0.5 CaCl_2_; pH 7.4, 340 to 350 mosmol/L. Horizontal slices were cut from the temporal part of the brain using a vibratome (VT1200S, Leica, Germany) at a thickness of 300 μm, corresponding to the ventral to mid‐dorsal portion of the hippocampus (Bregma −7.6 to −4.7 mm). Slices were then allowed to recover in sACSF at 32°C–34°C for 30 min in a submerged holding chamber. Subsequently, slices were kept at room temperature until recording. All chemicals used in the physiological solutions were purchased from Roth (Germany), Merck (Germany), or Sigma‐Aldrich (USA) unless otherwise stated.

### 2.2 Electrophysiology

Slices were transferred to a submerged recording chamber mounted on an upright microscope (BX51WI, Olympus, Japan). Continuous perfusion was maintained at a rate of 5 mL/min with carbogenated, prewarmed (32°C–34°C) ACSF containing (in mM): 125 NaCl, 2.5 KCl, 25 NaHCO_3_, 1.25 NaH_2_PO_4_, 25 glucose, 1 MgCl_2_, 2 CaCl_2_, 1 Na‐pyruvate, 1 ascorbic acid. Pipettes were pulled using borosilicate glass capillaries (Hilgenberg, Germany) using a horizontal puller (P‐97, Sutter Instruments, CA, USA). When filled with intracellular pipette solution (in mM): 135 K‐gluconate, 10 KCl, 2 MgCl_2_, 0.1 EGTA, 10 HEPES, 2 Na_2_‐ATP, 0.3 Na2‐GTP, 1 Na_2_‐creatinine, 0.1% biotinylated‐lysine (Biocytin, Invitrogen), the electrodes had a series resistance of 3–5 MΩ. Infrared differential interference contrast and a digital camera (RetigaTM ELECTRO, Teledyne QImaging, UK) were used to visualize and target neurons for whole‐cell patch clamp recordings. Subicular PYNs were targeted on the basis of their elongated oval‐ or triangular‐shaped soma and randomly targeted in Sub2 according to published landmarks (Ishihara et al. 2020; Ishihara, Miyamoto, et al. 2025; Ishihara, Sato, et al. 2025). For further PYN identification, the VGAT‐YFP fluorescent signal was assessed in a subset of the neurons under epifluorescent LED illumination (470 nm, pE‐100, CoolLED, UK). Only neurons that lacked a signal were considered PYN and selected for patching. Neurons were recorded in current clamp mode using a patch‐clamp amplifier (Axopatch 200B, Molecular Devices, CA, USA). Recorded data was filtered at 10 kHz and digitized at a sampling rate of 20 kHz using an analog‐to‐digital interface (PCIe‐6321 National Instruments, TX, USA). The open‐source software WinWCP (University of Strathclyde Glasgow, https://spider.science.strath.ac.uk) was utilized to record data for subsequent off‐line analysis in the open‐source software application Stimfit (https://github.com/neurodroid/stimfit).

Intrinsic active and passive membrane properties were assessed using current steps ranging from −250 to 250 pA in steps of 50 pA (500 ms duration) as previously described (Grosser et al. 2021; Guinet et al. 2024). The resting membrane potential (RMP) was derived from the average voltage response over a 50 ms baseline period prior to the application of a current step in the initial recording. The following intrinsic passive membrane properties were assessed from voltage responses to a −50 pA current pulses of 500 ms duration. Input resistance (Rin) was calculated from the voltage difference between the preceding baseline and the steady‐state response using Ohm's law. The apparent membrane time constant (tau) was derived by fitting a mono‐exponential curve to the decay of the response. To investigate active membrane properties, single APs elicited by smallest depolarizing current step producing discharge were analyzed. The value of the corresponding current was taken as the estimate of rheobase. AP amplitude was measured from the voltage threshold (i.e., when the first derivative (dV/dt) reaches 20 mV/ms), to the peak. AP rise and decay rates were derived from the maxima of dV/dt during the rising and decaying AP phases, respectively. Rise time was determined by linear interpolation between neighboring sampling points and refers to the time required for the signal to change from 20% to 80% of the peak value (measured from the baseline).

Afterhyperpolarization (AHP) is a negative deflection following an AP or at the end of a series of APs. Fast AHP (fAHP) was taken as the negative peak occurring immediately after an AP, measured from threshold. Medium AHP (mAHP) was taken as the second, slower negative peak after the AP. Slow AHP (sAHP) was measured as the peak of the prolonged negative deflection relative to the initial baseline after a series of APs, by a +250 pA current pulse. Maximal discharge frequency was derived from the 250 pA AP response. Voltage “sag” amplitude was measured in averaged responses (250 pA, 3 traces) between the initial negative peak and steady state during pulse application. To identify burst versus regular discharge, the interspike interval (ISI) ratio was taken from the second ISI to the first ISI in a pulse train elicited by a +250 pA current pulse. The latency of the first spike was calculated as the time between the onset of the current pulse and the threshold crossing of the first AP.

Neurons with a depolarized RMP lower than −50 mV or instability were excluded from analysis. As the series resistance is not a critical factor for the quality of the current‐clamp recordings using the Axopatch 700B amplifier, we accepted cells with up to 50 MΩ for this data set (range 5–50 MΩ).

### 2.3 Histology

Recorded slices were directly transferred to 4% paraformaldehyde containing solution with 0.1 mM phosphate buffer (PB) and 0.05% NaN_3_ and kept overnight at 4°C. Slices were then transferred to and rinsed repeatedly in 0.1 mM PB. Biocytin filled neurons were visualized using a streptavidin conjugated fluorochrome complex (Alexa Fluor, 647 nm, 1:1000, Invitrogen, USA; in 0.1 M PB with 10% Triton X‐100, Serva, Germany). Finally, slices were mounted on standard glass slides with 300 μM thick metal spacer to minimize shrinkage (Bolduan et al. 2020) and embedded using an aqueous mounting medium (Fluoromount‐G, Southern Biotech, USA).

Identity of all PYNs was confirmed after visualization on the basis of their morphological features: (1) characteristic somato‐dendritic morphology including an elongated or triangular shaped cell body, multiple basal dendrites emerging from the lower part of the soma and at least one apical dendrite with a distal tuft, (2) presence of spines densely covering the dendrites, and (3) lack of substantial axonal collateralization within the region.

To confirm location of the neurons within the subiculum, the layered cytoarchitecture of Sub2, corresponding to the proximal and middle parts, and the core‐like Sub1 according to Ishihara et al. (2020) were identified. In slices, in which the assessment of the cytoarchitecture was difficult on the basis of the background fluorescence labeling, we performed additional labeling for the perisomatic inhibitory marker parvalbumin (PV) (Booker et al. 2013). In brief, after rinsing the slices, a blocking step was performed in 10% normal goat serum (Gibco), including 1% Triton X‐100 (Serva) and 0.05% NaN3 in 0.1 mM PBS (1 h, room temperature). Slices were then incubated with a primary antibody (AB) against PV (polyclonal, guinea pig, 1:1000, Synaptic Systems, 2 days at 4°C). Following multiple rinsing steps in PBS, a fluorescent conjugated secondary AB was applied (anti‐guinea pig, 1:500, Invitrogen; 24 h 4°C). Subsequently slices were rinsed again and mounted as described above.

### 2.4 Confocal Imaging and Reconstruction

Visualized neurons were imaged using a confocal laser scanning microscope (FV1000, Olympus, Japan). High‐resolution image stacks (30× silicon immersion objective, N.A. 1.05, Olympus) of each neuron were captured for a full three‐dimensional reconstruction. FIJI software package (http://fiji.sc/) was employed to stitch image stacks, and reconstructions were performed using the semi‐automated neuron tracing software neuTube (https://neutracing.com). Image scaling, shrinkage correction (Bolduan et al. 2020) and measurement of morphological parameters were performed within the NEURON environment (Hines and Carnevale 1997) using custom scripts. For the analysis of branching patterns, Sholl profiles were generated using the Simple Neurite Tracer plug‐in of FIJI.

### 2.5 Anatomical Mapping of Recording Location

Location of each neuron within the subiculum was determined by obtaining low magnification (×4) overviews. To standardize the location of the neurons in the curved structure of the subiculum with varying transverse and vertical dimensions at different levels along the septo‐temporal axis, we linearized the subiculum and calculated the relative position between the anatomical boundaries of the region: The position of each neuron was measured from the CA1‐subiculum border (proximo‐distal position) and from the fissure (vertical position). These absolute distance values were normalized to the full length of the subiculum measured along the alveus and the vertical span between alveus and hippocampal fissure at the position of the neuron. Localization of all neurons was then mapped within this linearized and normalized coordinate representation of the subiculum. Neurons recorded in Sub1 were excluded from the analysis.

### 2.6 Hierarchical Clustering

Principal component analysis (PCA) was performed on subicular pyramidal neurons (*n* = 148) using MATLAB software package (2022b, MathWorks, USA). Since subicular PYNs have been classified by the timing and regularity of their APs upon synaptic stimulation or direct current injection, we based the PCA on their firing properties. Parameters used in the PCA were number of bursts at rheobase, number of APs within the initial burst, number of bursts at maximum depolarization (250 pA), and the number of APs within the initial burst at maximum depolarization, as well as first to third inter‐spike interval (ISI) and the ISI ratio. For PCA, data was standardized and subsequently clustered using an unsupervised hierarchical clustering algorithm (Ward's method).

### 2.7 Statistics

Data presented in this study is reported as mean ± standard error of mean (SEM) if not stated otherwise. The normality distribution of data was assessed using the Shapiro–Wilk normality test. For normally distributed data, two‐way ANOVA followed by Tukey's multiple comparison test was employed for pairwise comparisons. Kruskal–Wallis test with Dunn's post hoc comparison was used for non‐normal data distribution. To examine differences between two groups in normally distributed datasets of equal variance, an unpaired *t*‐test was applied, or data was corrected using Welch's test if variances differed. Non‐normal distributed data was tested using the Mann–Whitney *U* non‐parametric test. For all tests the significance levels indicated are: *p < 0.05, **p < 0.01, ***p < 0.001 and ****p < 0.0001.

## 3 Results

### 3.1 PCA Reveals Three Distinct Subicular PYN Subtypes Based on Discharge Pattern

**FIGURE 1:**
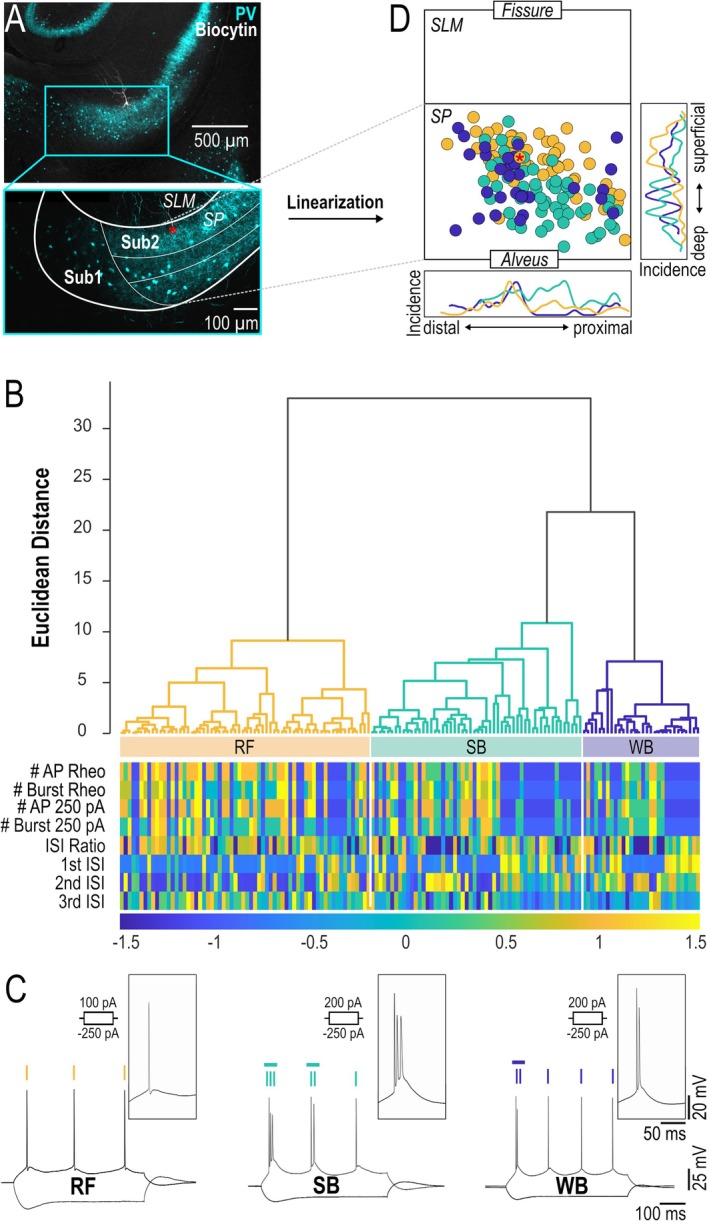
(A) Projected confocal image of hippocampal slice labeled for PV (in cyan) and a recorded and biocytin labeled PYN (white) in the subiculum. The lower panel shows a zoomed‐in image of the subiculum with delineating the two subregions, Sub1 and Sub2, and the layers of the Sub2. The position of the recorded neuron is labeled with a red asterisk. SLM, stratum lacunosum‐moleculare; SP, stratum pyramidale; Sub1, subiculum 1; Sub2, subiculum 2. (B) Dendrogram of the hierarchically clustered (Ward's method) high‐dimensional parameter space following PCA. Color coded bar below shows the z‐scores for all PCA parameters. Three distinct neuron types (RF, SB, WB) of a substantial Euclidean distance were found. (C) Representative voltage responses of each PCA assigned neuron type to de‐ and hyperpolarizing current steps. Insets depict the initial APs. (D) Recording location of each neuron with PCA assigned PYN subtype classification in a two‐dimensional, standardized and linearized representation of the Sub2. Red asterisk indicates the location of the neuron shown in (A). Diagrams on the right and below depict the incidence of RF, SB and WB neurons along the superficial‐deep (right) and proximo‐distal axis (below).

To investigate the electrophysiological and morphological characteristics of subicular PYNs, we conducted whole‐cell patch‐clamp recordings from a total of 148 PYNs (including 26 neurons from Guinet et al. 2024; see Methods). In this study, we focused on the Sub2, which shows a laminated structure and corresponds to the proximal and middistal parts of the subiculum (Ishihara et al. 2020). Neurons situated in the dense, core‐like distal portion of the subiculum (Sub1) were excluded from analysis. To determine the anatomical location of each neuron within the subiculum, cytoarchitectonic landmarks were used (Ishihara et al. 2020). As the perisomatic inhibitory marker PV has been shown to delineate cell body layers very well in the hippocampus, due to the localization of interneuron axons (Booker et al. 2013), we used this marker in order to assist the localization of the recorded neurons. Indeed, immunolabeling for PV was strong in the cell body layer of the Sub2, but weak in the Sub1 (Figure 1A). Hence, for a subset of neurons situated at the border between Sub1 and Sub2, cellular location was confirmed by PV staining.

First, we performed PCA using eight parameters that describe the discharge pattern of PYNs (Figure 1B). The following parameters were used: number of bursts at rheobase, number of APs within the initial burst, number of bursts at maximum depolarization (250 pA) and its corresponding number of APs within the initial burst, as well as first to third inter‐spike interval (ISI) and the ISI ratio (loadings of each variable are depicted in Figure S1B). As the first two principal components explained 75.7% of the total variance of the dataset, we separated branches of the obtained dendrogram of the subsequent hierarchically clustered data (Ward's method) at the second node. This analysis revealed three distinct groups of subicular PYNs of substantial Euclidean distance. The first group comprised RF neurons (*n* = 64, 43%), firing typically a single APs at threshold level (rheobase 116.4 ± 4.5 pA (Figure 1C). The second branch contained BF neurons, that could further be subdivided into strong bursting (SB, *n* = 54, 37%) and weak bursting (WB, *n* = 30, 20%) neurons. SB neurons discharged with two or more initial bursts (mean: 2.2 ± 0.1 bursts with 3.1 ± 0.1 APs per burst at rheobase, 131.5 ± 7.7 pA), whereas WB neurons fired one or two shorter bursts followed by single APs (mean: 1.6 ± 0.2 bursts with 2.0 ± 0 APs at rheobase, 125 ± 10.1 pA), Figure 1C, Table 1).

**TABLE 1:** Passive and active physiological properties of subicular PYN subtypes.

	RF (64)	SB (54)	WB (30)	*p*	
Passive properties					
Resting membrane potential [mV]	−62.63 ± 0.50	−61.69 ± 0.51	−61.55 ± 0.60	0.28	ns
Input resistance [MΩ]	170.2 ± 9.01	82.86 ± 3.15	86.37 ± 5.72	< 0.0001	****
Membrane time constant [ms]	22.18 ± 1.11	19.65 ± 1.05	22.94 ± 1.43	0.07	ns
Membrane capacitance [pF]	149.7 ± 9.2	254.1 ± 17.06	284.6 ± 21.51	< 0.0001	****
Active properties					
Rheobase [pA]	116.4 ± 4.46	131.5 ± 7.74	125 ± 10.1	0.44	ns
Sag amplitude [mV]	−7.86 ± 0.29	−5.32 ± 0.20	−5.28 ± 0.31	< 0.0001	****
AP rise time [ms]	0.23 ± 0.01	0.24 ± 0.01	0.22 ± 0.01	0.007	**
AP rise rate [mV/ms]	244.7 ± 8.42	246.6 ± 10.26	313.8 ± 19.34	0.006	**
AP decay rate [mV/ms]	72.56 ± 1.82	62.84 ± 2.36	77.33 ± 4.37	0.0008	***
Rise/decay ratio	3.37 ± 0.09	3.97 ± 0.13	4.08 ± 0.13	< 0.0001	****
Max discharge frequency [Hz]	23.05 ± 0.80	23.04 ± 0.77	21.8 ± 1.11	0.64	ns
1st interspike interval [ms]^a^	41.55 ± 2.29	5.19 ± 0.12	6.65 ± 0.52	< 0.0001	****
2nd interspike interval [ms]^a^	78.24 ± 2.86	9.26 ± 0.89	119.9 ± 4.78	< 0.0001	****
3rd interspike interval [ms]^a^	86.79 ± 3.08	150.6 ± 13.54	87.33 ± 9.08	< 0.0001	****
# APs per burst (rheo)^a^	0 ± 0	3.09 ± 0.07	2 ± 0	< 0.0001	****
# bursts (rheo)^a^	0 ± 0	2.24 ± 0.13	1.6 ± 0.17	< 0.0001	****
# APs per burst (250 pA)^a^	0.19 ± 0.07	2.94 ± 0.11	2.3 ± 0.12	< 0.0001	****
# bursts (250 pA)^a^	0.09 ± 0.04	1.72 ± 0.13	1.13 ± 0.12	< 0.0001	****
ISI ratio	0.55 ± 0.03	0.65 ± 0.02	0.06 ± 0.01	< 0.0001	****
AP Amplitude [mV]^b^	77.22 ± 1.26	76.56 ± 1.19	82.32 ± 1.59	0.04	*
AP half‐width [ms]	1.12 ± 0.02	1.26 ± 0.03	1.11 ± 0.06	0.002	**
Fast AHP amplitude [mV]^b^	−8.41 ± 0.54	0.55 ± 0.36	0.11 ± 0.58	< 0.0001	****
Medium AHP amplitude [mV]^b^	−10.95 ± 0.25	−6.56 ± 0.33	−8.87 ± 0.47	< 0.0001	****
Slow AHP amplitude [mV]^b^	−4.07 ± 0.23	−4.57 ± 0.19	−3.99 ± 0.24	0.18	ns

*Note:* Significance levels are indicated as *p < 0.05, **p < 0.01, ***p < 0.001 and ****p < 0.0001.

^a^ Included in PCA.

^b^ Measured from threshold.

Robustness of PCA was tested by reiterating multiple PCAs with a subset of the data. When excluding single neurons, independent of discharge pattern, all remaining neurons fell into their original clusters. Next, we randomly excluded 10%, 15%, and 20% of the dataset. For each pass, a few RF and SB neurons were assigned to the WB cluster, but divergence rates were consistently below 10% (5.3% for 10%, 3.2% for 15%, and 7.6% for 20% random data exclusion) and for each PCA three stable neuron groups of substantial Euclidean distance emerged (Figure S1C), verifying our primary clustering. Moreover, the total data variability captured by each principal component was strongly related for each PCA, also reflecting a high robustness of the original analysis (Figure S1D).

Differences in the spatial distribution of RF versus BF neurons have been previously suggested. Therefore, to analyze the spatial distribution of the three identified neuron classes, we mapped the PCA‐based neuron subtypes onto a linearized and normalized coordinate system of the Sub2 (Figure 1D; see Methods). This analysis revealed differential distribution of RF, SB, and WB neurons along the proximo‐distal and superficial‐deep axis. RF neurons were predominantly localized in the superficial cell body layer of Sub2 and hence exhibited a significantly smaller distance to the fissure than the two BF neuron subtypes (*p* < 0.0001; Table 2). In contrast, SB neurons were preferentially found in deeper layers of Sub2. Finally, WB neurons spread more homogeneously along the superficial‐deep axis. Along the proximo‐distal axis, RF and WB neurons were more prevalent in the distal Sub2 whereas SB neurons were scattered more homogeneously (Figure 1D).

**TABLE 2:** Morphometric properties of subicular PYN types.

	RF (60)	SB (52)	WB (30)	*p*	
General properties					
Distance to SLM [μm]	57.46 ± 5.73	143.2 ± 8.06	135.3 ± 14	< 0.0001	****
Total dendritic length [mm]	5.66 ± 0.24	7.25 ± 0.25	6.76 ± 0.27	< 0.0001	****
Axonal length [mm]	2.78 ± 0.20	2.28 ± 0.15	2.44 ± 0.23	0.2	ns
Soma diameter [μM]	15.57 ± 0.38	17.29 ± 0.36	16.73 ± 0.55	0.006	**
Soma vertical‐axis [μM]	21.1 ± 0.57	21.69 ± 0.45	21.91 ± 0.91	0.52	ns
Apical dendrites					
Length APD [mm]	3.52 ± 0.16	3.84 ± 0.18	3.58 ± 0.20	0.41	ns
Length apical tuft [mm]	2.40 ± 0.15	2.58 ± 0.18	2.38 ± 0.19	0.75	ns
Length oblique branches [mm]	0.99 ± 0.08	1.04 ± 0.09	1.03 ± 0.09	0.85	ns
Length apical main stem [mm]	0.15 ± 0.02	0.22 ± 0.02	0.17 ± 0.02	0.009	**
Horizontal extend [μm]	274.8 ± 9.86	277.1 ± 11.25	279.9 ± 13.71	0.97	ns
Vertical extend [μm]	490.4 ± 13.67	572.7 ± 11.82	503.8 ± 16.00	< 0.0001	****
# Proximal intersections	6.76 ± 0.31	4.63 ± 0.22	5.10 ± 0.37	< 0.0001	****
# Distal intersections	3.81 ± 0.36	5.22 ± 0.44	5.38 ± 0.70	0.002	*
Basal dendrites					
Number of primary BD	4.88 ± 0.24	5.77 ± 0.20	4.73 ± 0.20	0.0003	***
Length BD [mm]	2.14 ± 0.13	3.42 ± 0.14	3.19 ± 0.21	< 0.0001	****
Horizontal extend [μm]	217.4 ± 9.96	257.8 ± 7.14	251.1 ± 9.73	< 0.0001	****
Vertical extend [μm]	208.6 ± 8.63	225.3 ± 9.09	235.5 ± 11.37	0.12	ns
# Intersections	13.88 ± 0.70	21.98 ± 0.88	19.32 ± 1.12	< 0.0001	****

*Note:* Significance levels are indicated as *p < 0.05, **p < 0.01, ***p < 0.001 and ****p < 0.0001.

### 3.2 Electrophysiological Diversity of PCA Assigned Subicular PYN Subtypes

**FIGURE 2:**
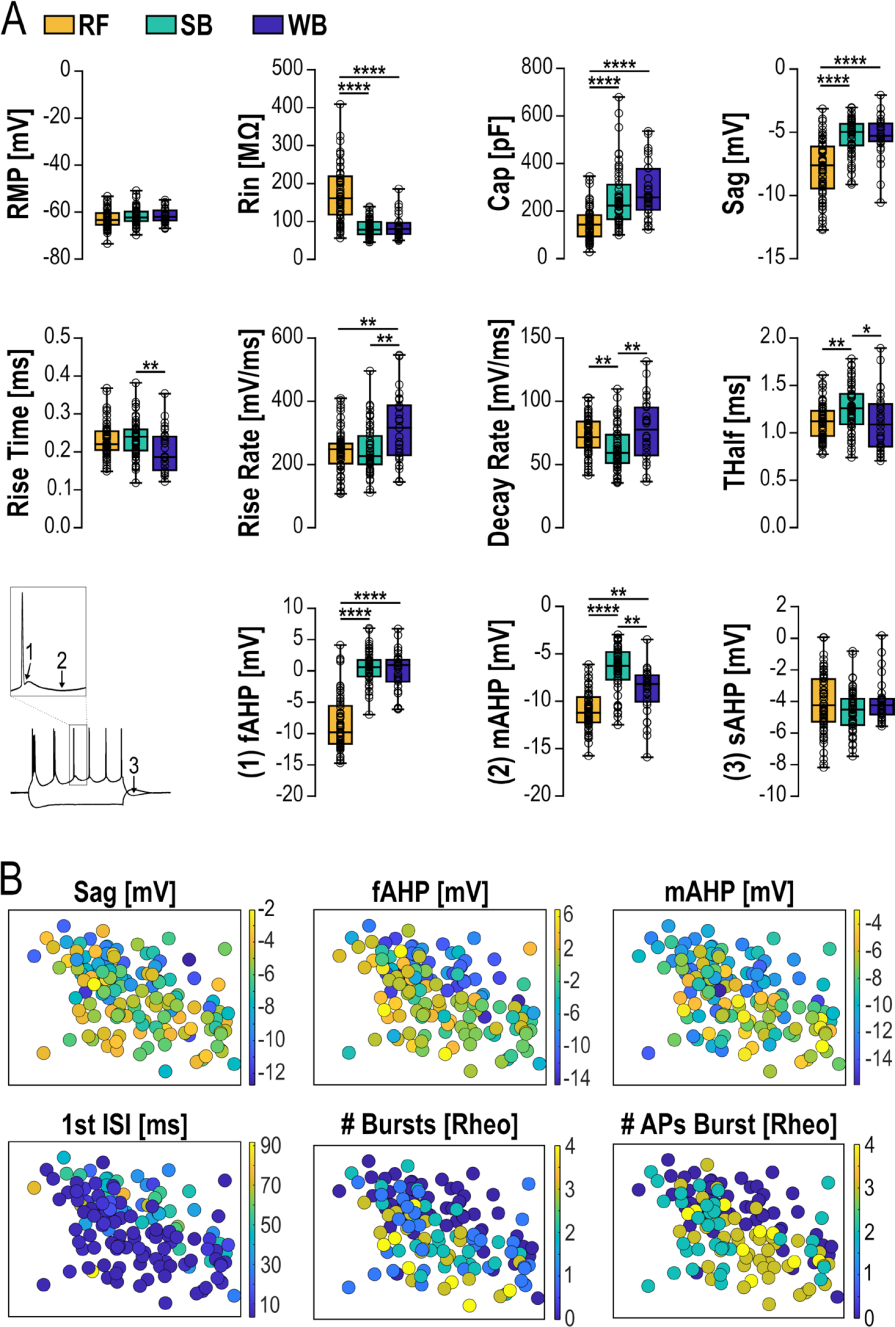
(A) Electrophysiological parameters of subicular PYN subtypes. Data is illustrated as box (25th to 75th percentile) with individual values superimposed. Differences were tested for significance using one‐way ANOVA with post hoc multiple comparison (normal distribution) or Kruskal–Wallis test with Dunns test. Significance levels were set to *p < 0.05, **p < 0.01, and ****p < 0.0001. (B) Spatial distribution of the neurons with significantly different electrophysiological parameters is illustrated on the linearized map of the Sub2. Color code shows the absolute values of the parameters.

We used the PCA assigned classification of Sub2 PYNs to investigate electrophysiological characteristics between the three subtypes and identified significant differences in passive and active membrane properties. RF neurons exhibited a 50% higher input resistance than WB or SB neurons (*p* < 0.0001) and a considerably lower capacitance (*p* < 0.0001). Divergence was also observed in subthreshold conductance, as RF neurons displayed larger sag potentials (at −250 pA) compared to either BF neuron subtype (*p* < 0.0001). Furthermore, RF neurons exhibited a more pronounced fast (*p* < 0.0001) and medium AHP (*p* < 0.0001) compared to BF neurons. Differences were also observed between the two BF neuron subtypes when we analyzed single AP waveforms (Figure 2A, Table 1). WB neurons exhibited a steeper AP rise rate (*p* < 0.01) and, thus, a significantly faster AP rise time (20%–80%, p < 0.01) compared to SB neurons. In addition, the AP decay rate of WB neurons was higher (*p* < 0.001), resulting in a shorter AP half height‐width compared to SB neurons (*p* < 0.05). WB neurons also exhibited a significantly faster medium AHP than SB neurons (Figure 2A, Table 1). Finally, the AP peak amplitude of WB neurons was larger (*p* < 0.05) compared to both SB and RF neurons (Figure 2A, Table 1).

When electrophysiological features, such as fast (fAHP) and medium after hyperpolarization (mAHP) or number of bursts and number of APs in the initial burst, were allocated onto the standardized subiculum for each neuron, we observed spatial distributions corresponding well to the distribution pattern of the subtypes identified by the PCA (Figure 2B).

### 3.3 Distinct Morphological Features of Subicular PYN Subtypes

**FIGURE 3:**
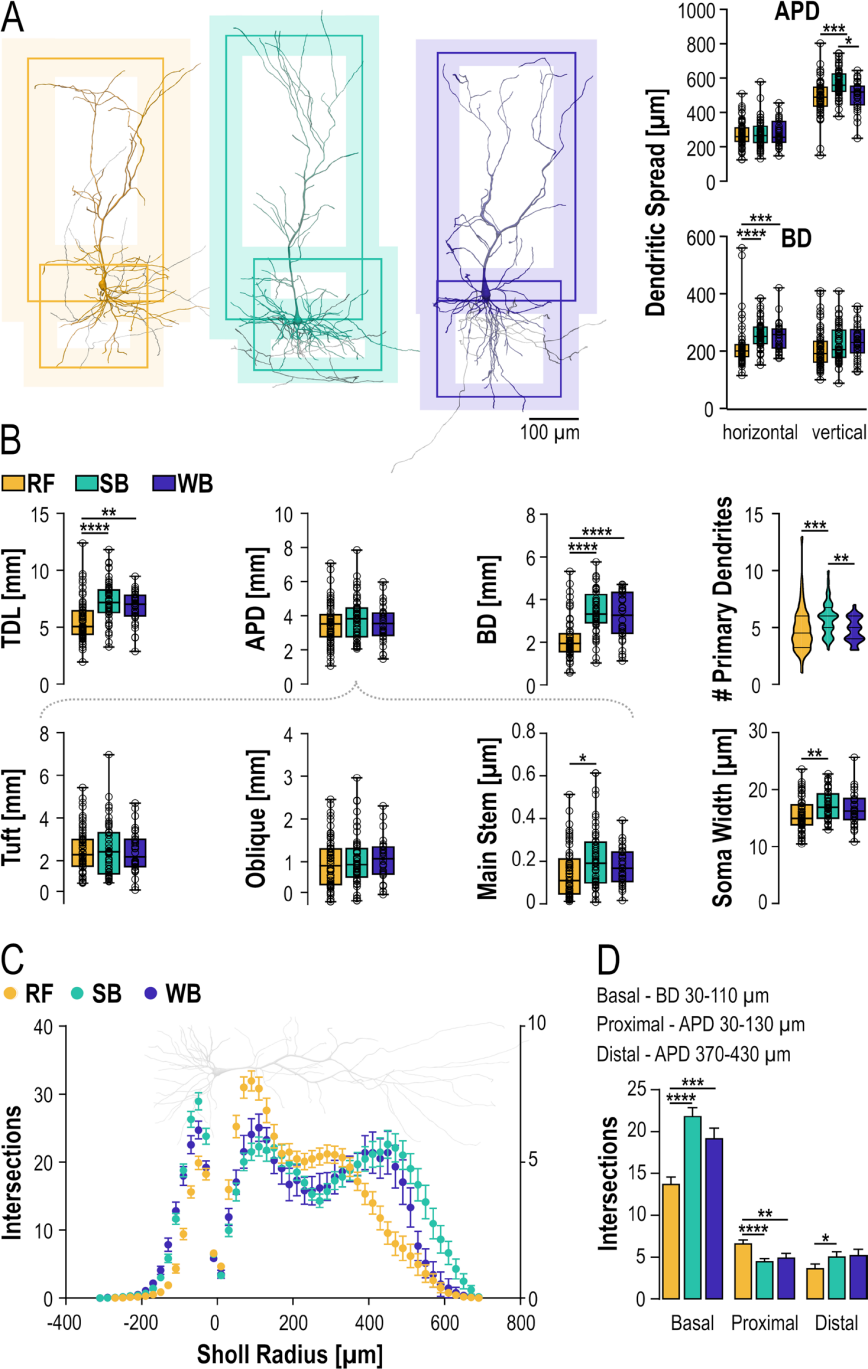
(A) Representative reconstructions of RF, SB and WB neurons. Boxes illustrate the average vertical and horizontal extent of basal and apical dendrites (shaded region corresponds to the 25th to 75th percentile). (B) Morphometric comparison of dendritic subdomains of the three PYN subtypes, with summary box plots of the total dendritic length (TDL), length of apical and basal dendrites (APD and BD), the length of the apical tuft and oblique dendrites, as well as the main apical stem. (C) Basal and apical dendritic Sholl profiles of the three subicular PYN subtypes. (D) Quantification of dendritic Sholl intersections (mean ± SEM) within three ranges corresponding to the basal dendrites, the proximal apical (oblique branches) and the distal apical (tuft) dendritic domains. Data was tested for significance using one‐way ANOVA with post hoc multiple comparison (normal distribution) or Kruskal–Wallis test with Dunns test. Significance levels were set to *p < 0.05, **p < 0.01, ***p < 0.001 and ****p < 0.0001.

Next, we assessed the somato‐dendritic morphology of PYN subtypes. Neurons exhibiting incomplete morphology, such as substantially cut dendrites, were excluded from the analysis. We first examined the spatial spread of the basal and apical dendrites of the individual PYNs from all three neuron groups (60 RF, 52 SB, and 30 WB neurons). RF neurons were consistently smaller than both WB and SB neurons. They exhibited a smaller horizontal spread of their basal dendrites (*p* < 0.0001), as well as a smaller vertical spread of their apical dendrites (*p* < 0.0001) (Figure 3A, Table 2). RF neurons also exhibited a significantly shorter total dendritic length than WB and SB neurons (*p* < 0.0001). Interestingly, this difference in total dendritic length was largely due to a shorter basal dendrite (*p* < 0.0001), whereas the length of the apical dendrites was comparable between the subtypes despite the differences in their vertical spread (Figure 3B). Only the length of the apical main stem, but not the oblique side branches or the apical tuft, differed significantly between RF and SB neurons (*p* < 0.01). In fact, the length of the apical main stem co‐varied with the depth of the neurons, and the more superficial localization of RF neurons versus the deeper localization of SB neurons corresponded well to the difference in the length of the apical stem between these two subtypes (Figure S2A–C, apical main stems of Devil cells were averaged for this analysis). Interestingly, SB neurons also showed a higher number of primary basal dendrites (*p* < 0.001) compared to RF and WB neurons. Finally, when analyzing the soma size, we found that while the subtypes had comparable vertical dimension, RF neurons had significantly smaller diameter of the cell body (*p* < 0.006, Figure 3B, Table 2).

To compare branching complexity between groups, we performed Sholl analysis for the three PYN subtypes (Figure 3C,D, Table 2). In line with the lower number of primary dendrites and the smaller basal dendritic length, RF neurons displayed a significantly lower number of basal dendritic intersections within a radius of 30–110 μm from the soma (*p* < 0.0001). Since RF neurons also exhibit shorter apical main stems, we found a more pronounced branching for the proximal domain (30–130 μm, *p* < 0.0001) and a less pronounced branching for the distal domain of their apical dendrites (Figure 3C,D).

Since subicular PYNs project out of the cortex and target other subcortical areas, and only a limited part of the axonal distribution is preserved in the slices, we did not perform a quantitative analysis of the axon. Nevertheless, we analyzed the origin of the axon. For 10.1% of the neurons, the axon emerged from a dendrite: in 12 of these neurons from a basal dendrite (5 RF, 4 SB, and 3 WB neurons) and in three neurons from the apical dendrite (2 WB and 1 SB neuron). The average distance of the axon initial segment from the dendritic origin was 3.3 ± 0.7 μm.

### 3.4 A Distinct Morphological Type of Subicular RF Neuron

**FIGURE 4:**
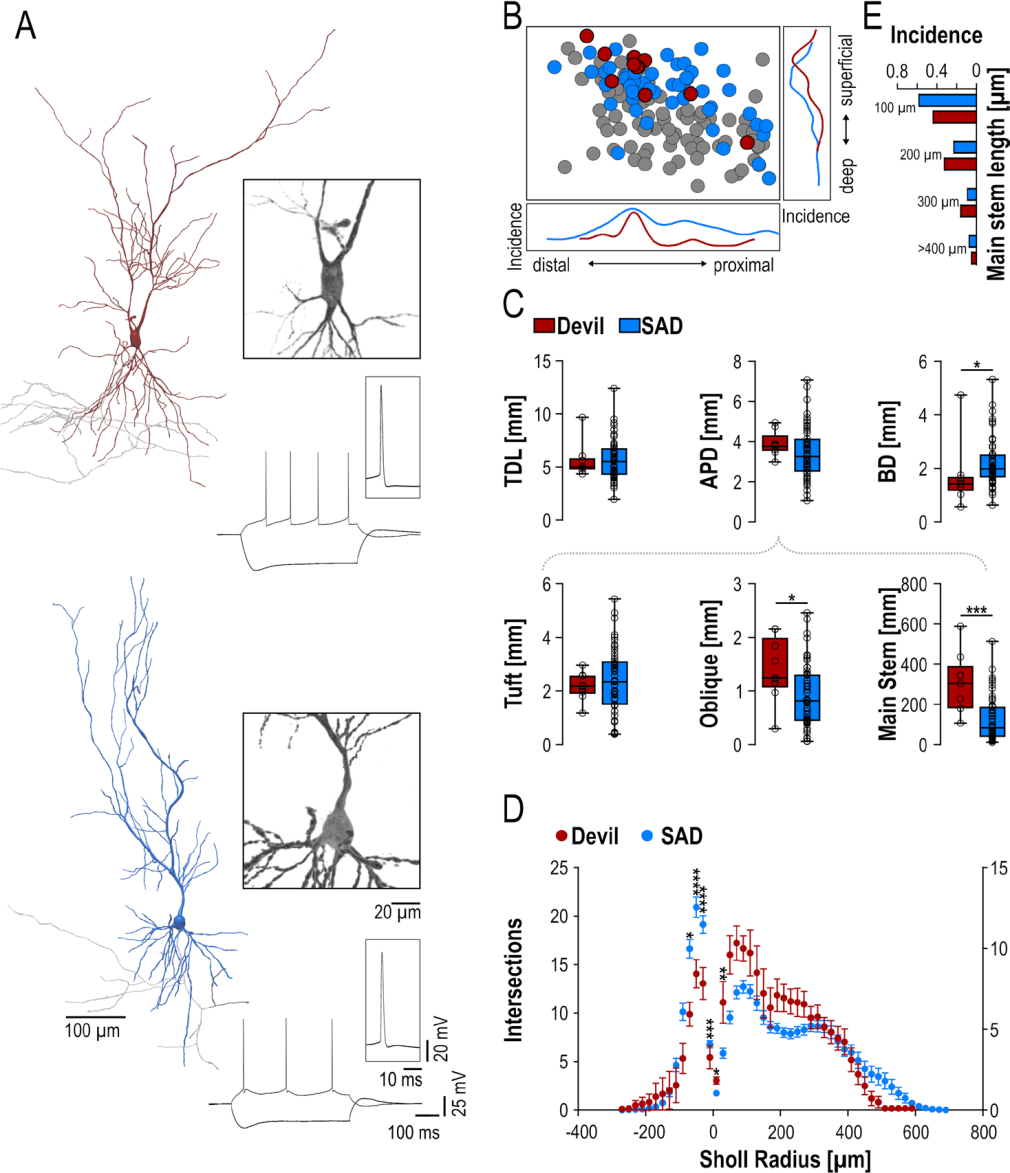
(A) Representative reconstructions of a Devil and a SAD RF neurons. Insets on the right: High‐power inverted images of the soma and proximal dendrites (top) of the two neurons; single action potentials (middle) and voltage responses of the two neurons to large de‐ and hyperpolarizing current pulses. (B) Distribution of RF neurons within the Sub2, with SAD drawn in blue and Devil RF neurons in red (WB and SB cells in gray). Diagrams on the right and below depict the incidence of Devil cells and SAD neurons along the superficial‐deep (right) and proximo‐distal axis (below). (C) Summary box plots of dendritic parameters of SAD und Devil RF neurons: the total dendritic length (TDL), the length of apical and basal dendrites (APD and BD), the apical tuft and oblique dendrites, as well as the main apical stem. (D) Sholl profiles of SAD und Devil RF neurons. Significance was assessed by using unpaired *t*‐test (normal distribution) or Mann–Whitney test. Significance levels indicated are *p < 0.05, **p < 0.01, ***p < 0.001, and ****p < 0.0001. (E) Histogram showing the normalized distribution of the main stem lengths for individual apical dendrites of Devil and SAD neurons.

Our morphological analysis further revealed PYNs with distinct morphology among RF neurons. These neurons exhibited two apical main stems emerging from two sides of a typically inverted triangular shaped soma (“Devil cells”), instead of the usual singular axial apical dendrite (SAD, Figure 4A). This distinct morphological subtype has been described in other hippocampal regions; however, they constituted a small subset and localized often outside the cell body layer and were therefore classified as displaced *PYNs* of CA1 and CA2 (Gulyás et al. 1998; Helton et al. 2019; Jarsky et al. 2008). This morphological subtype has not been described and characterized in the subiculum yet. In our sample, *Devil cells* represent 7.4% of PYNs (*n* = 11 of total 148 PYNs) and their cell body was consistently found in the cell body layer. Notably, all Devil cells recorded were RF; none was found among SB and WB neurons. These PYNs, thus, consistently discharged with trains of single APs upon depolarization (Figure 4A) and represented 17% of all RF neurons. Electrophysiologically, Devils cells and RF neurons with a single apical dendrite (SAD) exhibited comparable properties (Table 3), and their spatial distribution corresponded also well to each other (Figure 4B). Morphologically, however, we found subtle differences between these two RF groups beyond the number of main apical dendrites. Neurons exhibiting incomplete morphology were excluded from this morphometric analysis, leaving 9 Devil cells and 51 SAD RF neurons for this analysis. Devil cells featured less primary basal dendrites (*p* < 0.05) with a smaller horizontal spread (*p* < 0.01) and a shorter total length (p < 0.05) compared to SAD RF neurons (Figure 4C, Table 4). In good agreement, Sholl profiles of basal dendrites showed a more complex branching for SAD RF neurons within the first 100 μm around the soma (Figure 4D). In contrast, Devil cells exhibited a higher branching complexity of the apical dendrites with a longer apical main stem (the two apical dendrites summed, *p* < 0.001) and a higher total length of oblique branches compared to SAD neurons (Figure 4C, *p* < 0.5). This finding was further reflected in a higher number of intersections 50–250 μm apically from the soma in the Sholl profile (Figure 4D). The distribution of main stem lengths for each individual apical dendrite was comparable between groups (Figure 4E).

**TABLE 3:** Passive and active physiological properties of SAD and Devil cells.

	Devil (11)	SAD (53)	*p*	
Passive properties				
Resting membrane potential [mV]	−61.75 ± 1.11	−62.81 ± 0.55	0.42	ns
Input resistance [MΩ]	174.8 ± 14.29	174.6 ± 10.41	0.28	ns
Membrane time constant [ms]	18.89 ± 2.22	22.87 ± 1.25	0.20	ns
Membrane capacitance [pF]	145.0 ± 24.69	150.7 ± 9.97	0.82	ns
Active properties				
Rheobase [pA]	131.8 ± 12.2	113.2 ± 4.70	0.12	ns
Sag amplitude [mV]	−7.85 ± 0.34	−7.85 ± 0.46	0.93	ns
AP rise time [ms]	0.22 ± 0.02	0.23 ± 0.01	0.52	ns
AP rise rate [mV/ms]	241.0 ± 8.48	262.9 ± 27.60	0.35	ns
AP decay rate [mV/ms]	73.53 ± 5.34	72.36 ± 1.92	0.81	ns
Rise/decay ratio	3.58 ± 0.32	3.33 ± 0.08	0.79	ns
Max discharge frequency [Hz]	21.52 ± 1.60	23.37 ± 0.91	0.53	ns
AP Amplitude [mV]^a^	78.36 ± 3	76.98 ± 1.36	0.5	ns
AP half‐width [ms]	1.11 ± 0.03	1.13 ± 0.06	0.82	ns
Fast AHP amplitude [mV]^a^	−8.37 ± 0.62	−8.59 ± 0.98	0.96	ns
Medium AHP amplitude [mV]^a^	−11.02 ± 0.25	−10.59 ± 0.87	0.64	ns
Slow AHP amplitude [mV]^a^	−4.01 ± 0.27	−4.33 ± 0.45	0.61	ns

^a^ Measured from threshold.

**TABLE 4:** Morphometric properties of subicular RS neurons.

	Devil (9)	SAD (51)	*p*	
General properties				
Total dendritic length [mm]	5.56 ± 0.54	5.67 ± 0.27	> 0.9999	ns
Axonal length [mm]	2.53 ± 0.18	2.82 ± 0.24	0.55	ns
Soma X‐axis [μM]	15.25 ± 1.08	15.63 ± 0.41	0.72	ns
Soma Y‐axis [μM]	22.28 ± 1.9	20.89 ± 0.58	0.37	ns
Apical dendrites				
Length APD [mm]	3.87 ± 0.40	3.46 ± 0.18	0.36	ns
Length apical tuft [mm]	2.17 ± 0.17	2.40 ± 0.17	0.58	ns
Length oblique branches [mm]	1.40 ± 0.20	0.92 ± 0.08	0.06	*
Length apical main stem [mm]	0.30 ± 0.05	0.13 ± 0.02	0.0004	***
Horizontal extend [μm]	278.7 ± 33.21	274.1 ± 10.19	0.87	ns
Vertical extend [μm]	497.0 ± 9.42	561.6 ± 72.55	0.79	ns
Basal dendrites				
Number of primary BD	3.67 ± 0.44	5.00 ± 0.27	0.05	*
Length BD [mm]	1.69 ± 0.40	2.22 ± 0.13	0.01	*
Horizontal extend [μm]	205.4 ± 45.4	219.5 ± 8.88	0.009	**
Vertical extend [μm]	302.8 ± 143.8	217.5 ± 9.2	0.06	ns

*Note:* Significance levels are indicated as *p < 0.05, **p < 0.01 and ***p < 0.001.

## 4 Discussion

In this study, we identified three electrophysiological subtypes of PYNs in the Sub2 by employing unsupervised clustering on parameters linked to discharge properties. These subtypes converged in their electrophysiological and morphometric features, indicating a functional and structural specialization of PYN subsets in Sub2. We further identified *Devil cells*, an additional fourth subtype, as a morphological subset of RF neurons. Finally, our results reveal a differential spatial distribution of PYN subtypes along the transverse and vertical axes of the Sub2.

### 4.1 Classification of PYNs in Sub2 Into Three Functional Subtypes

Prior studies have classified PYNs of the subiculum into electrophysiological subtypes based on discharge phenotypes (Behr et al. 1996, 2009; Graves et al. 2012; Kim and Spruston 2012; Taube 1993; Wozny et al. 2008). In this study, we employed parameters linked to firing behavior to perform PCA to obtain an unbiased functional classification. Subsequent unsupervised clustering identified three stable groups of subicular PYNs of considerable Euclidean distance: RF, SB and WB neurons. Our PCA differentiated SB and WB neurons within the BF population, converging with the results of Staff et al. (2000). In contrast to Menendez de la Prida et al. (2003), however, we could not find a second functional subtype among RF neurons. Thus, we reveal and confirm distinct electrophysiological subicular subtypes in an unbiased manner by one of the first approaches of unsupervised, hierarchical clustering of subicular PYNs.

Consistent with their classification as PYNs, intrinsic electrophysiological properties were largely comparable between groups, as described earlier (Staff et al. 2000; Taube 1993; Wozny et al. 2008). However, we also observed differences in passive membrane properties between subicular PYN subtypes, such as the higher input resistance and lower capacitance of RF compared to BF neurons (Jarsky et al. 2008; Kim and Spruston 2012; Menendez de la Prida 2003), indicating a higher excitability of RF PYNs. We also found distinct variations in active properties: AP kinetics and afterpotentials between groups. Fast and medium AHPs were substantially smaller in BF neurons compared to RF cells, as reported earlier (Behr et al. 1996; Guinet et al. 2024; Kim and Spruston 2012; Staff et al. 2000). Our results revealed further, unreported differences between the two BF subtypes. Maximal rise and decay rates of APs were the highest, the AP amplitudes the largest, and the AP half‐width the shortest in WB neurons compared to RF and SB neurons. Hence, WB neurons are not an intermediate subtype between RF and SB neurons, rather, they constitute a distinct physiological subtype.

### 4.2 Morphometric Differences Among Subicular PYN Subtypes

Morphologically, subicular PYNs have been described as a largely homogeneous population with similar morphometric features of apical and basal dendrites across the subiculum (Graves et al. 2012; Matsumoto et al. 2019). However, in addition to the electrophysiological differences, we found substantial morphologic divergence, resulting in distinct morphometric characteristics of the three subicular PYN subtypes. Most prominently, RF PYNs had a substantially lower dendritic length as well as horizontal and vertical spread than both WB and SB neurons. This difference was mostly due to a lower number of primary dendrites, lower branching and length of the basal dendrites of RF. As the basal dendrites are the main site of recurrent excitatory inputs, this difference in basal dendritic architecture suggests distinct roles of RF and BF neurons in the subicular network with RF neurons getting less local synaptic input than BF neurons. This is in good agreement with the electrophysiological observations that connectivity among subicular PYNs is directed from RF to BF cells (Böhm et al. 2015). In the apical dendritic tree, only the main stem of RF cells was shorter than in the other two subtypes. These results can be explained by the more superficial localization of RF PYNs, as it has been previously observed that the first major apical branching point of subicular PYNs is at around the upper boundary of *stratum pyramidale* (Matsumoto et al. 2019). Finally, the difference in the dendritic length between RF and BF neurons may, at least partially, explain the difference in their physiological phenotypes: First, a larger dendritic surface of BF PYNs may accommodate a larger contingent of calcium channels underlying their burst firing discharge pattern (Joksimovic et al. 2017; Jung et al. 2001). Second, the difference in the dendritic tree, as well as the smaller soma size correlate well with the higher input resistance and lower rheobase of RF neurons.

While the apical and basal dendritic length and spread of SB and WB neurons was comparable, SB neurons exhibited a higher number of primary basal dendrites than WB neurons. However, since we found no difference in the total basal dendritic length between SB and WB neurons, the lower number of primary basal dendrites seems to be compensated by more branching and, overall, longer basal dendrites in WB neurons, which likely reflects similar synaptic input.

### 4.3 Distinct RF Morphological Subtypes

Our morphological analysis furthermore revealed a specific subtype among subicular PYNs. Harris and colleagues (Harris et al. 2001) have previously referred to a few neurons, bearing two apical dendrites at the superficial edge of the subicular PYN layer. However, they haven't provided a thorough characterization of electrophysiological or morphological features of these neurons. Our data show that these “*Devil cells”* are all RF PYNs and their intrinsic active and passive properties are comparable to SAD RF cells. However, morphologically, Devil cells exhibited further differences, including longer apical main stems with a higher branching and higher length of oblique side branches. In contrast, the basal dendritic length was significantly smaller compared to SAD RF neurons. Those morphometric variations between both subclasses were also reflected in their Sholl profile and could indicate a higher specialization of Devil cells as target of cross‐regional connections in the subicular network versus afferent inputs.

### 4.4 Distribution of Subicular PYN Subtypes and Their Possible Functional Roles

When we mapped electrophysiological PYN types onto a standardized and linearized subiculum, the emerging spatial pattern revealed a differential distribution of PYN subtypes along the vertical and proximo‐distal axes of Sub2: RF neurons, both SAD and Devil cells, were preferentially located more superficially in the cell body layer, with increasing density toward the distal part of Sub2. The two BF PYN subtypes, in contrast, were situated in deeper layers. However, while SB neurons were spread homogeneously along the proximo‐distal axis, WB neurons showed a preference for the distal part of Sub2. However, there was no difference observed in the spatial distribution between the two RF subsets. A number of previous studies have shown complementary localization for RF and BF cells along the vertical (Greene and Totterdell 1997; Matsumoto et al. 2019) or the proximo‐distal axes (Cembrowski, Wang, et al. 2018; Jarsky et al. 2008). Our study, thus, confirms and reconciles these observations, identifying divergent two‐dimensional rules for the distinct morpho‐electric PYN subtypes.

The distinct spatial organization of subicular PYN subtypes underlines specific functional roles of RF and BF neurons within the subicular network, as suggested earlier (Behr et al. 1996; Cembrowski, Phillips, et al. 2018; Kim and Spruston 2012; Menendez de la Prida et al. 2003; Wozny et al. 2008). Our results show that superficially situated RF neurons have the highest excitability, as reflected by their high input resistance and low rheobase. While their basal dendritic arbor is smaller, indicating that they receive less local recurrent excitation, in good agreement with observations from multi‐electrode circuit analysis (Böhm et al. 2015), their proportionally large apical dendrites reach out to *stratum lacunosum‐moleculare* to receive synaptic input from CA1 and the medial entorhinal cortex. Therefore, RF PYNs are likely to be recruited first by these major afferents, especially if the input is weak. As perisomatic inhibition and inhibitory plasticity are stronger in RF cells (Guinet et al. 2024), their activity will remain under tight inhibitory control. Increasing afferent input will next recruit BF neurons, further supported by an overflow of excitation from the active RF population via the unidirectional recurrent excitatory connectivity (Böhm et al. 2015). Due to the stronger recurrent connectivity and weaker inhibition, as well as their bursting discharge, the recruitment of BFs may rapidly become non‐linear at increasing excitation levels and result in a shift in the dominance of population activity from RF to BF PYNS. As RF and BF neurons project to different target structures (Cembrowski, Phillips, et al. 2018; Kim and Spruston 2012), such a shift will bring about a change in the direction of information flow out of the hippocampus. The impact of the subtle differences in the physiological and morphological features between WB and SB neurons within this context remains open and requires further investigation. The subicular organizational rules of its discrete functional PYN subtypes presented in this study will be critical for the understanding of memory encoding and retrieval in the healthy and pathological brain.

## Funding

This work was supported by the Deutsche Forschungsgemeinschaft (CRC‐TRR 384/1 TP B07, 514483642).

## Conflicts of Interest

The authors declare no conflicts of interest.

## Supporting information


**Figure S1:** (A) Primary PCA of Sub2 PYNs, depicted again for reference. (B) Loadings of each variable for the first eight principal components. (C) Robustness test of the PCA by repeating the analysis with subsets of the original data: First, a single neuron of either type (RF, SB, and WB, black tick) was excluded (top bars). Second, we randomly excluded 10%, 15%, and 20% of the dataset (black ticks). For each pass, a few RF and SB neurons (red ticks) were assigned to the WB cluster, but divergence rates from the original remained below 10% (5.3% for 10%, 3.2% for 15%, and 7.6% for 20%). (D) Total data variability captured by each principal component depicted for the PCA iterations.

**Figure S2:** (A) Histogram showing the distribution of apical stem lengths within the population of subicular PYNs (50 μm bins). The length of apical main stems of Devil cells was averaged for this analysis. (B) Representative reconstructions of subicular PYNs with different lengths of apical stems (color code: apical stem length, as in panel A). (C) Linearized subicular map depicting the spatial distribution of the neurons with the length of the apical main stem (color code as in A). Note that neurons with shorter apical main stems are typically situated superficially.

## Data Availability

The data that support the findings of this study are available from the corresponding author upon reasonable request.
