# Supplementary figures and images for: Spatial Organization of Morpho‐Electric Subtypes of Pyramidal Neuron in the Subiculum
Source: Hippocampus. 2026 Feb 16;36(2):e70081. doi: 10.1002/hipo.70081 (PMC12908100; doi:10.1002/hipo.70081)

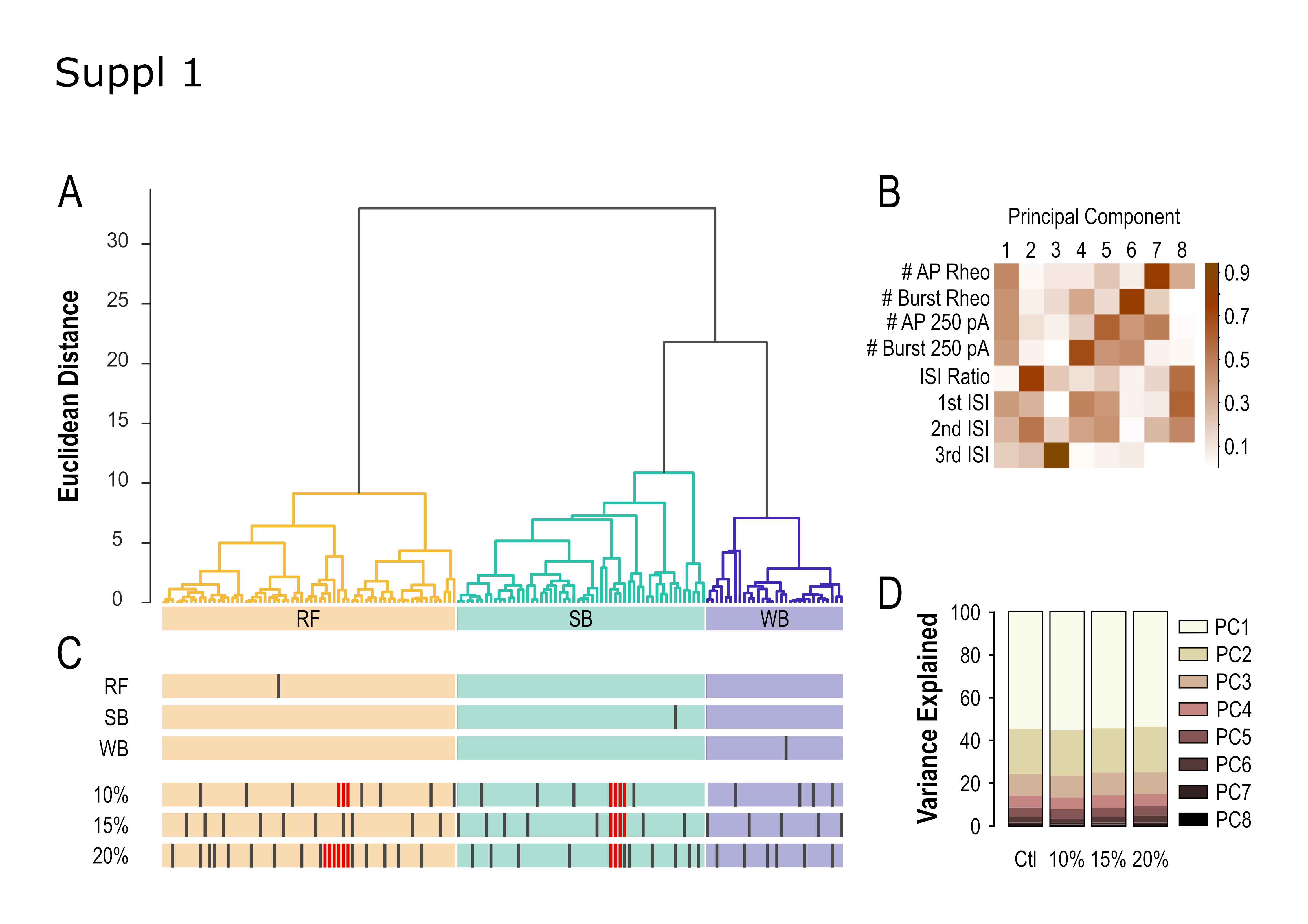

Supplement: Supplementary file 1 — Figure S1: (A) Primary PCA of Sub2 PYNs, depicted again for reference. (B) Loadings of each variable for the first eight principal components. (C) Robustness test of the PCA by repeating the analysis with subsets of the original data: First, a single neuron of either type (RF, SB, and WB, black tick) was excluded (top bars). Second, we randomly excluded 10%, 15%, and 20% of the dataset (black ticks). For each pass, a few RF and SB neurons (red ticks) were assigned to the WB cluster, but divergence rates from the original remained below 10% (5.3% for 10%, 3.2% for 15%, and 7.6% for 20%). (D) Total data variability captured by each principal component depicted for the PCA iterations. [file HIPO-36-0-s002.png]

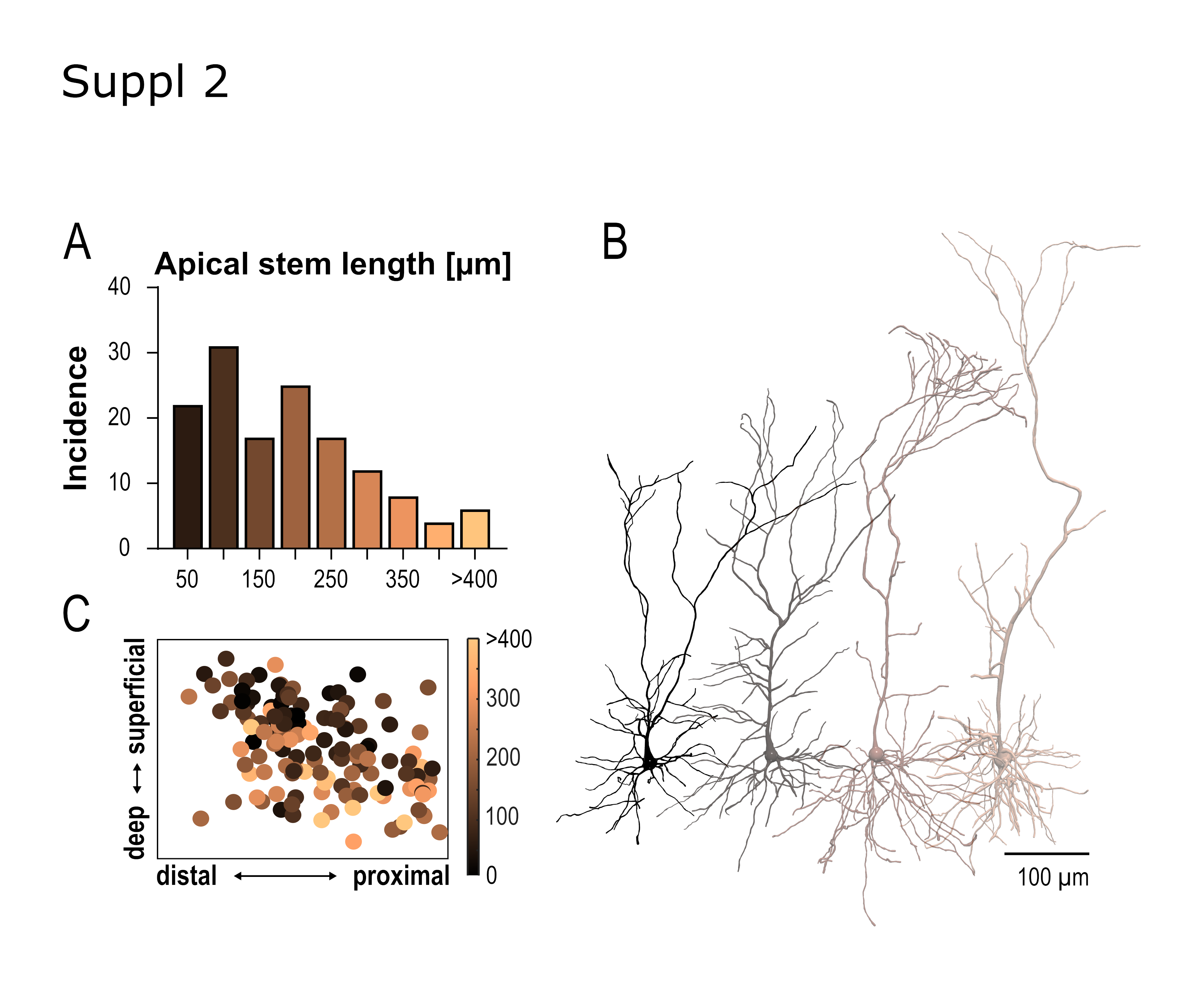

Supplement: Supplementary file 2 — Figure S2: (A) Histogram showing the distribution of apical stem lengths within the population of subicular PYNs (50 μm bins). The length of apical main stems of Devil cells was averaged for this analysis. (B) Representative reconstructions of subicular PYNs with different lengths of apical stems (color code: apical stem length, as in panel A). (C) Linearized subicular map depicting the spatial distribution of the neurons with the length of the apical main stem (color code as in A). Note that neurons with shorter apical main stems are typically situated superficially. [file HIPO-36-0-s001.png]
